# Supplementary figures and images for: Physical Activity Patterns among Individuals with Prediabetes or Type 2 Diabetes across Two Years—A Longitudinal Latent Class Analysis
Source: Int J Environ Res Public Health. 2022 Mar 19;19(6):3667. doi: 10.3390/ijerph19063667 (PMC8949382; doi:10.3390/ijerph19063667)

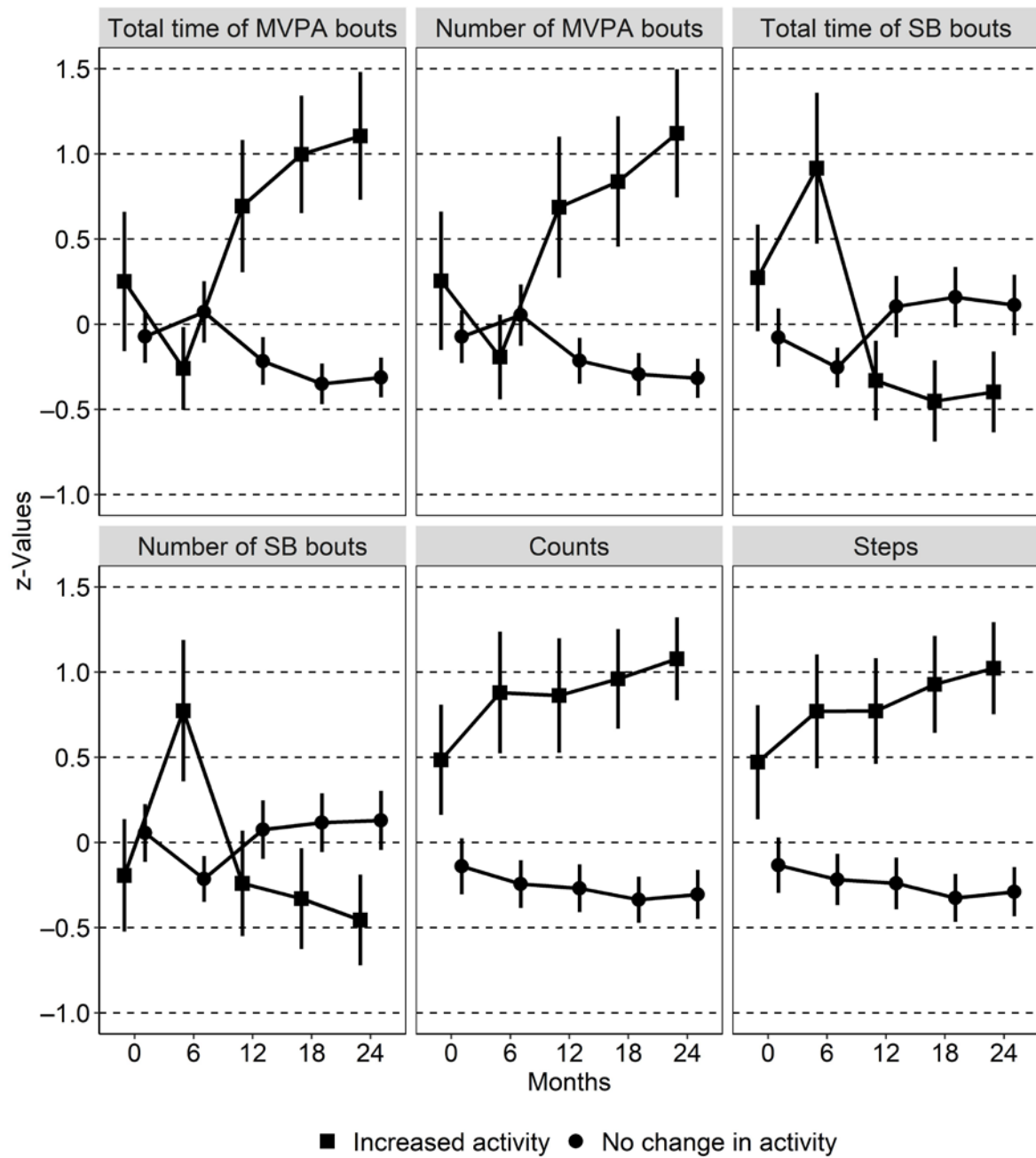

Figure S1.

Supplement: Supplementary file 1 [file ijerph-19-03667-s001.zip › ijerph-1617463-supplementary.pdf]
